# Evidence for non-neutralizing autoantibodies against IL-10 signalling components in patients with inflammatory bowel disease

**DOI:** 10.1186/1471-2172-15-10

**Published:** 2014-02-28

**Authors:** Natalie Frede, Erik-Oliver Glocker, Jennifer Wanders, Karin R Engelhardt, Wolfgang Kreisel, Frank M Ruemmele, Bodo Grimbacher

**Affiliations:** 1 Centre of Chronic Immunodeficiency, University Medical Centre Freiburg, Engesser Straße 4, 79108 Freiburg, Germany; 2 Department of Immunology, Division of Infection and Immunity, University College London, Royal Free Hospital, London, UK; 3 Institute of Medical Microbiology and Hygiene, University of Freiburg, Freiburg, Germany; 4 Department of Gastroenterology, Hepatology and Endocrinology, University Hospital Freiburg, Freiburg, Germany; 5 Université Paris-Descartes, Sorbonne, Paris Cité. INSERM U989, Assistance Publique-Hopitaux de Paris, Hôpital Necker Enfants Malades, Service de Gastroentérologie pédiatrique, Paris, France

**Keywords:** Inflammatory bowel disease, Autoantibodies, Autoimmunity, Interleukin-10, IL-10 receptor

## Abstract

**Background:**

Inflammatory bowel disease constitutes a heterogeneous group of conditions, whose aetiology is only partly understood. The prevailing hypothesis on its pathogenesis is that IBD is the result of an inadequate immune response to the resident bacterial flora of the intestine. An autoimmune background, however, has been discussed since the 1950s. Lately, it has been shown that failures in interleukin-10 (IL-10) signalling due to IL-10- and IL-10 receptor (IL-10R) mutations result in IBD. Our study aimed at investigating the existence of inhibitory autoantibodies against IL-10 and IL-10R in IBD patients capable of down-modulating IL-10 signalling thereby mimicking IL-10 or IL-10R deficiency.

**Results:**

Thirteen IBD patients had IgG autoantibodies against IL-10, IL-10RA and/or IL-10RB, and three patients had IgA autoantibodies against IL-10. However, the absolute OD values of the serum antibodies measured by ELISA were low, there was overall no significant difference between patients and controls, and positive sera had no neutralizing activity.

**Conclusion:**

No evidence for an involvement of autoantibodies against IL-10 or IL-10R in the pathogenesis of inflammatory bowel disease could be established.

## Background

Inflammatory bowel disease (IBD) includes Crohn’s disease, ulcerative colitis and indeterminate colitis and is characterized by a chronic and relapsing inflammation of the small or large intestine, abdominal pain, diarrhea, bleeding and malabsorption. With a prevalence of up to 450 per 100,000 inhabitants in the UK, IBD constitutes a common inflammatory condition; hence, the impact of associated morbidity and mortality is estimated to be substantial [1].

IBD is considered as the result of an excessive and inadequate immune response to commensals of the intestine, defining IBD as a pro-inflammatory disorder [2]. In addition, at least in a subgroup of patients, IBD might also be an immunodeficiency due to impaired release of pro-inflammatory cytokines by macrophages [3,4]. This hypothesis is supported by several primary immunodeficiencies that are characterized by IBD-like phenotypes such as IPEX syndrome, XIAP deficiency, and NEMO deficiency [5-7]. IL-10- and IL-10R deficiencies add to these disorders: affected patients manifest with severe early-onset enterocolitis which shows a dramatic and life-threatening progress [8-10]. The latter underscore the necessity of IL-10 to control inflammation and keep our immune system in balance [11-13].

For a long time, an autoimmune background has been suggested for IBD, and antibodies against numerous auto-antigens have been detected [14-18]. In 2009, Ebert et al. described autoantibodies against various cytokines in IBD patients leading to a state of relative deficiency of IL2, TGF-β and IL-10 [19]. Given the relevance of IL-10 as the key immunomodulatory factor of the human immune system, neutralizing autoantibodies against IL-10 or IL-10R might significantly impair IL-10 signalling and contribute to the severity of IBD.

In this study, we aimed to investigate the existence of autoantibodies binding to IL-10 or to the IL-10R, thus abrogating IL-10 signalling and mimicking a state of IL-10 deficiency. Here, we present the results of a screening for autoantibodies of the IgG and IgA isotype, putatively directed against IL-10 signalling components in 52 IBD patients.

## Results and discussion

### Antibodies against IL-10

#### Antibodies of the IgG isotype

**Figure 1:**
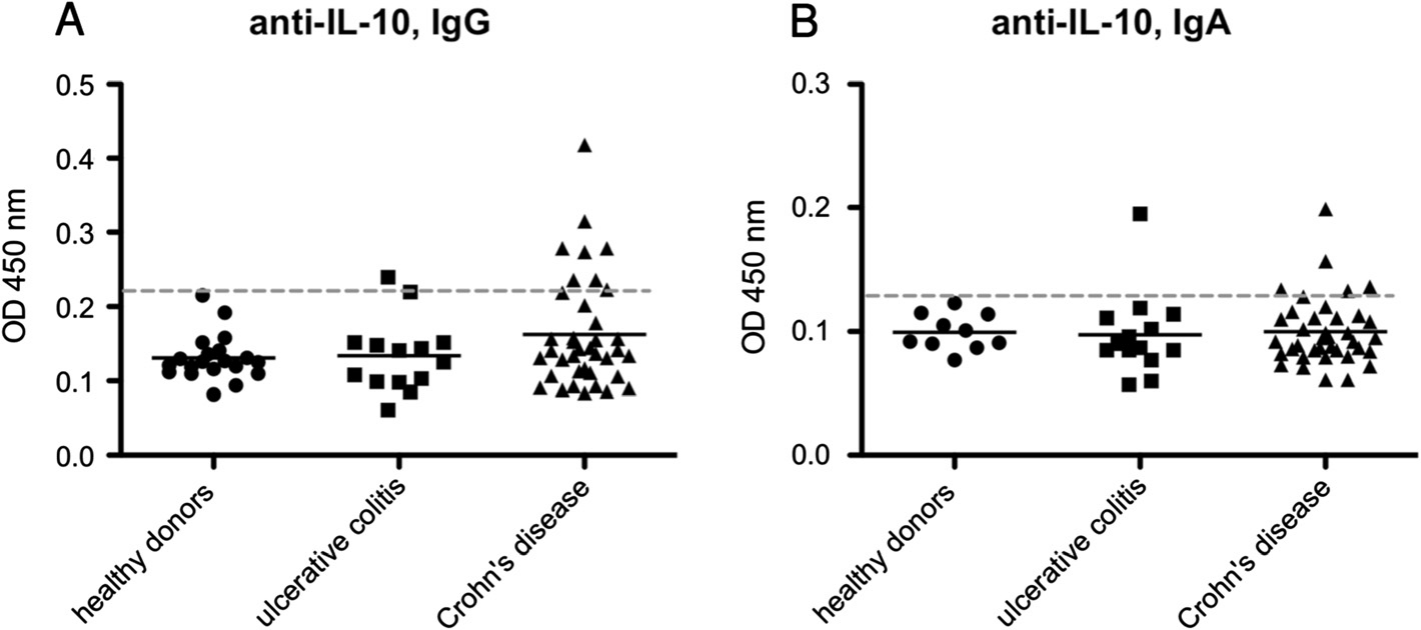
**Autoantibodies against IL-10.** Serum samples of patients suffering from inflammatory bowel disease were tested for anti-IL-10 Immunoglobulin G **(A)** and Immunoglobulin A **(B)** by ELISA. The dotted line represents the cut-off, whereas means are depicted as solid lines.

Sera of 20 healthy donors as well as 52 IBD patients were subjected to ELISA testing in search of anti-IL-10 IgG antibodies. Upon testing, eight out of 52 IBD patients (15%) showed OD values above a cut-off of μ + 3SD (Figure 1A). Data were analyzed by t-test, comparing the Crohn’s disease (CD) and ulcerative colitis (UC) groups both to the healthy control group. We found the CD group to exhibit significantly elevated levels of anti-IL-10 IgG antibodies, whereas the UC group testing resulted in a non-significant p-value (p = 0.41). In our cohort, 18% of CD patients (7/38) had anti-IL-10 autoantibodies; this replicates the results of Ebert et al. [19], who found 17% of CD patients to have anti-IL-10 IgG autoantibodies in the serum, thus validating our results in spite of a small sample size.

However, it has also been reported that anti-IL-10 antibodies may be present in high titers in healthy blood donors [20,21], and anti-cytokine autoantibodies are believed to occur ubiquitously in healthy individuals [22]. Furthermore, in contrast to other diseases with neutralizing anti-cytokine autoantibodies [23,24], but similar to the anti-IL-10 antibodies found by Ebert et al. [19], the increase in OD values was not striking in our patients.

#### Antibodies of the IgA isotype

Previous studies only reported on IgG anti-IL-10 antibodies in their populations. However, IgA constitutes a crucial element of mucosal immunity and is the most abundant immunoglobulin in the gut. Thus, its putative role in IBD has often been speculated upon and early reports implied that abnormal IgA function may play a role in the pathogenesis of IBD [25].

We therefore extended our search for the detection of anti-IL-10 autoantibodies of the IgA isotype. The sera of 52 IBD patients, i.e. 38 CD and 14 UC patients, and 10 randomly chosen healthy donors were probed.

In three patients (2 CD and 1 UC), elevated ODs were found for anti-IL-10 IgA autoantibodies, while means of all groups were not significantly different (Figure 1B).

The number of patients with anti-IL-10 IgA autoantibodies might however, be underestimated with the chosen assay. Probing of serum samples may not be the best method to detect autoantibodies of the IgA isotype, as antibodies may accumulate in the inflamed tissue of the gut so that concentrations significantly higher than in the serum may be reached. Also, while IgA makes up two thirds of the produced antibodies, it is mostly secreted so that it constitutes only 15% of the immunoglobulins present in human serum. Additionally, it is possible that the titre of autoantibodies fluctuates over time in a given patient.

In conclusion, our results suggest that single IBD patients might produce class IgA autoantibodies against IL-10. Yet, the significance of these antibodies is unclear.

### Antibodies against the IL-10R

#### Antibodies of the IgG isotype

**Figure 2:**
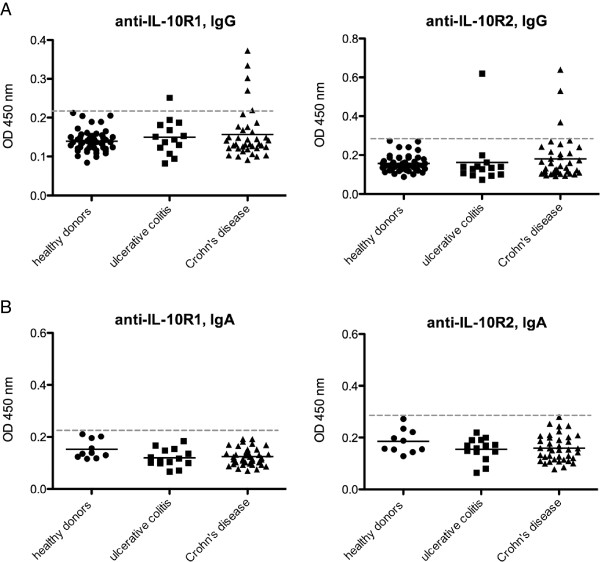
**Autoantibodies against the IL-10 receptor.** Serum samples of IBD patients and healthy donors were tested by ELISA for IgG **(A)** and IgA **(B)** antibodies binding to IL-10R1 and IL-10R2. The dotted line represents the cut-off, whereas means are depicted as solid lines.

In order to investigate whether IBD patients might produce IgG antibodies binding to the IL-10 receptor, sera of 50 healthy blood donors and 52 IBD patients were analysed by ELISA for binding to either recombinant IL-10R1 or IL-10R2 (Figure 2A).

Neither for anti-IL-10R1 nor for anti-IL-10R2 significant differences between means of IBD patients and healthy blood donors could be detected; statistical analysis failed to reach the significance level of p < 0.05. Stratifying patients according to the site of disease manifestation did not change the results (Additional file 1: Figure S1).

However, few patients proved to have significantly elevated ODs of up to 4 times the mean suggesting that some IBD patients do produce autoantibodies against IL-10R1 or IL-10R2. In the CD group, three out of 38 patients (8%) tested for anti-IL-10R2 had values that were higher than any value measured in the healthy donor group (method after [19]), compared to one out of 14 patients (7%) in the UC group. Regarding anti-IL-10R1, four out of 38 CD patients (11%) and one out of 14 UC patients (7%) had elevated values.

Another method to determine a cut-off for positive values is to calculate μ + 2SD or μ + 3SD, i.e. the mean of a healthy donor population and addition of 2 or 3 standard deviations. Using the μ + 3SD method on our samples provided exactly the same results as the method described above, i.e. one UC and four CD patients were above the calculated cut-off for anti-IL-10R1 and one UC and three CD patients for anti-IL-10R2. Thus, IgG autoantibodies against the IL-10R seem to be produced by a non-significant number of patients in our cohort.

#### Antibodies of the IgA isotype

The sera of 52 IBD patients, i.e. 38 CD and 14 UC patients, and 10 randomly chosen healthy donors were probed for anti-IL-10R autoantibodies of the IgA isotype.

No increased absorption values were found for anti-IL-10R1 and anti-IL-10R2 IgA autoantibodies in patients’ sera (Figure 2B), indicating that neither Crohn’s disease nor ulcerative colitis patients from our cohort produce IgA autoantibodies against the IL-10R.

#### Limitations of the ELISA technology as a screening method

Recombinant proteins, such as the rIL-10R1 and rIL-10R2, may have different epitopes than the *in vivo* receptor on the cell surface. Hence, antibodies binding to the rIL-10R might in fact not recognize the IL-10 receptor *in vivo* and *vice versa*. Furthermore, it is well known that surface-protein interactions, e.g. between the ELISA plate and the recombinant protein, can induce conformational changes and therefore subsequently inhibit or facilitate antibody binding. In order to address these limitations, a functional assay was developed to evaluate the ELISA results.

#### Functional test for neutralizing activity of anti-IL-10R antibodies

To assess whether the detected IL-10R autoantibodies have functional consequences, their impact on STAT3 phosphorylation, a key event in IL-10 signalling, was determined.

**Figure 3:**
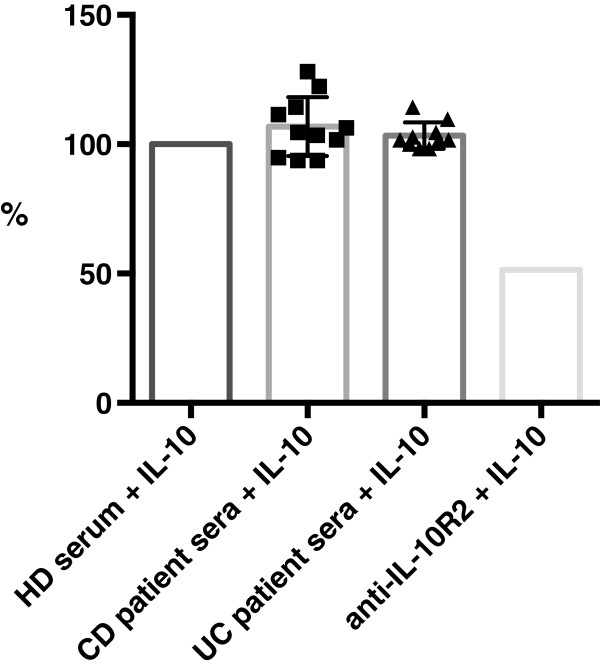
**Functional test assessing STAT3 phosphorylation.** PBMCs were incubated with healthy donor (HD) or patients’ sera in a 1:10 dilution and subsequently stimulated with IL-10. Cells were lysed and examined for STAT3 phosphorylation by ELISA. An anti-IL10R2 antibody was used as a positive control. The OD of the healthy donor control was set as 100%.

Sera of UC and CD patients (n = 21) were compared with serum of a healthy donor control. A commercial antibody directed against IL-10R2 (R&D, UK) was used as a positive control. Analysis of STAT3 phosphorylation upon IL-10 stimulation of PBMCs pre-incubated with a 1:10 dilution of serum failed to demonstrate a significant reduction of ODs, and therefore STAT3 phosphorylation, in the IBD patient cohorts (Figure 3). There were no significant differences between ELISA-positive and -negative samples or between UC and CD samples.

In conclusion, we could not find evidence for the functional relevance of autoantibodies against the IL-10 receptor. Neither did we find any evidence for the existence of autoantibodies, which were not detected by ELISA but had a sufficiently high titer to significantly reduce downstream signalling.

### Single and multiple positives

In total, 13 of 52 patients (25%) were found to have autoantibodies against IL-10 or the IL-10R subunits IL-10R1 and IL-10R2 (Table 1).

**Table 1:** Patients single or multiple positive for antiautobodies against the IL-10 signalling pathway

***N***^***o ***^ ***of patients with antibodies against:***	**IL-10**	**IL-10R1**	**IL-10R2**	**IL-10 + IL-10R1**	**IL-10 + IL-10R2**	**IL-10 + IL-10R1 + IL-10R2**
*IgG*	3	2	1	1	2	1
*IgA*	2	-	-	1^1^	-	-
*Total n*^*o*^*of patients*	5	2	1	2	2	1

^1^This patient had anti-IL-10 antibodies of the IgG and IgA isotype.

Thirteen patients had autoantibodies affecting the IL-10 signalling pathway. Eight patients had antibodies against a single protein, and five patients had antibodies against multiple components.

Whereas eight patients had antibodies against either IL-10 or one subunit of the IL-10R alone, sera of four patients were double positive for antibodies against IL-10 and IL-10R; one patient had serum triple positive for anti-IL-10, anti-IL-10R1 and anti-IL-10R2 antibodies. The latter patients might generally have an increased auto-immune status with possibly other autoantibodies in the serum as well. This, the low absolute OD values, and the fact that the IL-10R autoantibodies had no neutralizing activity, suggest that the autoantibodies might not be of overall significance.

## Conclusion

Our results do not provide sufficient evidence to postulate a general autoimmune disruption of IL-10 signalling in the pathogenesis of IBD.

Overall, no significant difference could be detected between IBD patients and healthy controls, with only singleton patients having been tested positive for autoantibodies against IL-10 and/or IL-10R in serum. Furthermore, the absolute OD values of detected autoantibodies were low with only a minor increase above the cut-off level in most positive patients. Most significantly, the sera positive for anti-IL-10R antibodies failed to show neutralizing activity in a functional test, further supporting the assumption that the detected antibodies are of little significance. Lastly, the finding that some patients were double or even triple positive for autoantibodies against IL-10 and IL-10R might reflect a low level autoimmune state with a broader range of autoantibodies with unknown significance.

While the understanding of the molecular mechanisms underlying inflammatory bowel disease gradually increases, further research needs to be conducted in order to identify the exact immune processes contributing to the complex pathogenesis of IBD. Genetic and functional data suggest IBD might be even more heterogeneous than initially expected [26,27]. Only after these topics have been addressed, more specific targeted therapies can be developed.

## Methods

### Patient cohorts

Patients’ serum samples (n = 52) were collected from IBD clinics in Freiburg and Paris, Necker Enfants Malades. Thirty-eight patients had Crohn’s disease and 14 had ulcerative colitis. Patients were diagnosed according to the Consensus of the European Crohn’s and Colitis Organisation (ECCO) [28,29] or the Porto-criteria and classified according to the Paris classification [30,31]. Samples were aliquoted upon receipt. All patients gave their informed consent; approval was granted by the National Research Ethics Service Committee London – Hampstead, formerly North West London REC 2/ Royal Free Hospital and Medical School REC, London, UK (Ref. 08/H0720/46 for healthy donors and 04/Q0501/119 for patients).

### Enzyme-linked immunosorbent assay (ELISA)

Flat-bottomed microtitre plates (Nunc-Immuno Plate Maxisorp Surface) were coated with recombinant IL-10R1 (R&D Systems) or IL-10R2 (Abnova) at a concentration of 2 μg/ml or with 0.5 μg/ml IL-10 diluted in carbonate buffer, pH 9.5 and incubated overnight at 4°C. The plates were washed with PBS-Tween (0.05%). Free binding sites on the plate were blocked with 300 μl 1% bovine serum albumin (BSA) (Sigma, US) for an incubation time of 1 h.

Human sera were added in a 1:200 dilution. Following a 2 h incubation period the plates were washed 5 times*.* The secondary antibody was added and incubated for 1 h. After washing, 100 μl of substrate TMB were added to each well. Colour development was monitored and 50 μl of 2 M sulphuric acid were added to stop the reaction. Absorption was determined in an ELISA reader at 450 nm.

### STAT3 phosphorylation assay

Peripheral blood mononuclear cells (PBMCs) were isolated using the Ficoll technique and then washed in PBS (Sigma, US). 10^5^ PBMCs were resuspended in 180 μL Opti-Mem I serum-free medium (Invitrogen, UK), added in 96-well plates and then pre-incubated for one hour with 20 μL neat or 1:10 diluted serum from anti-IL-10R auto-antibody positive patients. Cells were then stimulated with 5 ng IL-10 (R&D, UK) for 10 minutes, lysed and examined for STAT3 phosphorylation by ELISA (Cell signaling, UK) according to manufacturer’s instructions.

## Abbreviations

IBD: Inflammatory bowel disease; CD: Crohn’s disease; UC: Ulcerative colitis; IL-10: Interleukin 10; IL-10R: IL-10 receptor; APECED: Autoimmune polyendocrinopathy-candidiasis-ectodermal dystrophy; CMC: Chronic mucocutaneous candidiasis.

## Competing interests

The authors declare that they have no competing interests.

## Authors’ contributions

NF carried out the ELISA and STAT3 phosphorylation assays. EOG designed the study and drafted the manuscript. JB carried out ELISA assays. KRE participated in the design of the study and helped to draft and revise the manuscript. WK diagnosed patients and collected serum samples. FMR diagnosed patients, collected serum samples and did the subgroup analysis of Crohn’s disease patients from Paris. BG conceived of the study, and participated in its design and coordination and helped to write the manuscript. All authors read and approved the final manuscript.

## Supplementary Material

Additional file 1: Figure S1 Subgroup analysis of Crohn’s disease patients from Paris. Crohn’s disease patients were subgrouped for disease localisation after their sera were tested for IgG (A) and IgA (B) autoantibodies against IL-10 and IgG autoantibodies against the IL-10 receptor (C) by ELISA. The dotted line represents the cut-off, whereas means are depicted as solid lines. Click here for file
