# Supplementary figures and images for: Evidence for non-neutralizing autoantibodies against IL-10 signalling components in patients with inflammatory bowel disease
Source: BMC Immunol. 2014 Feb 28;15:10. doi: 10.1186/1471-2172-15-10 (PMC3942769; doi:10.1186/1471-2172-15-10)

**Supplementary Figure S1**


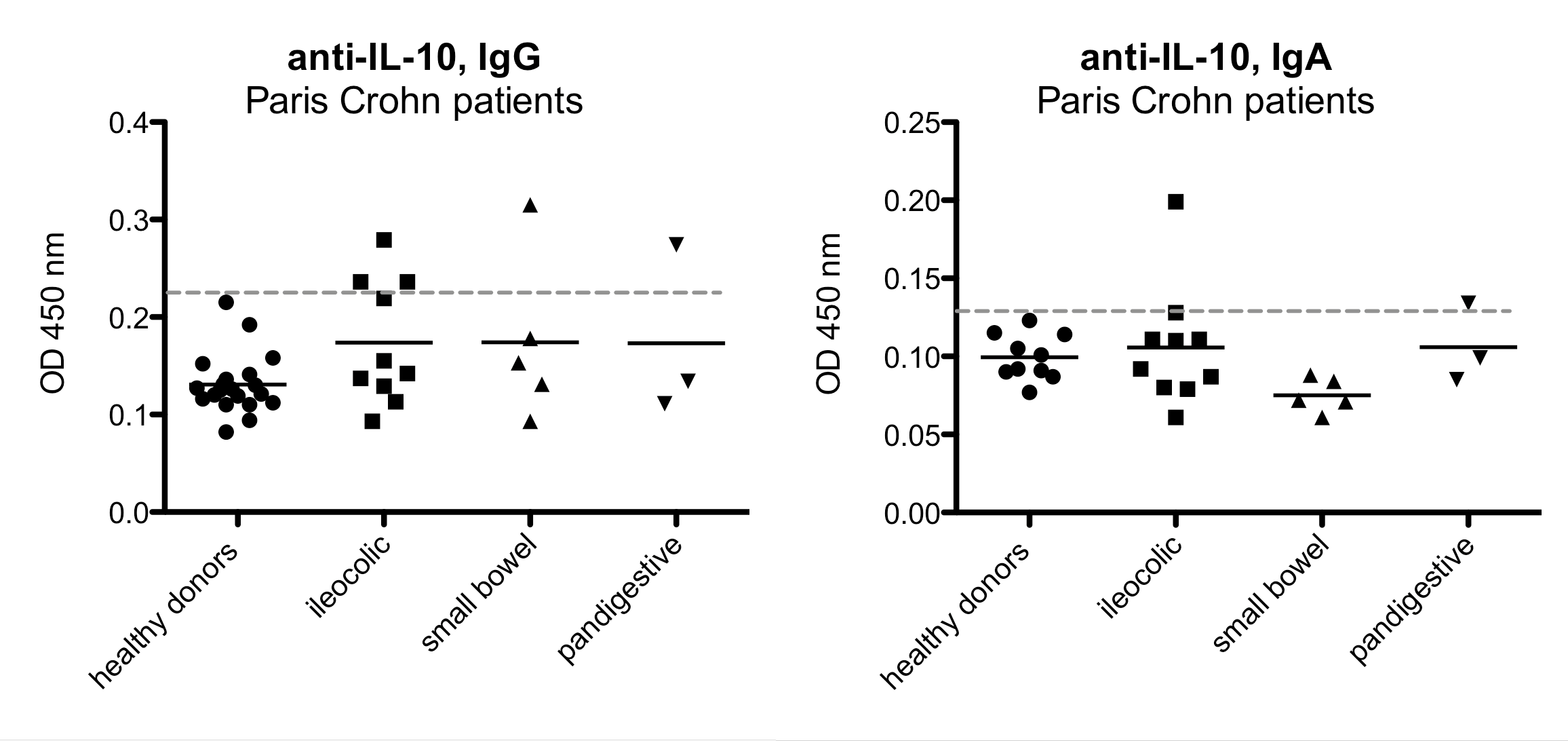


A

B


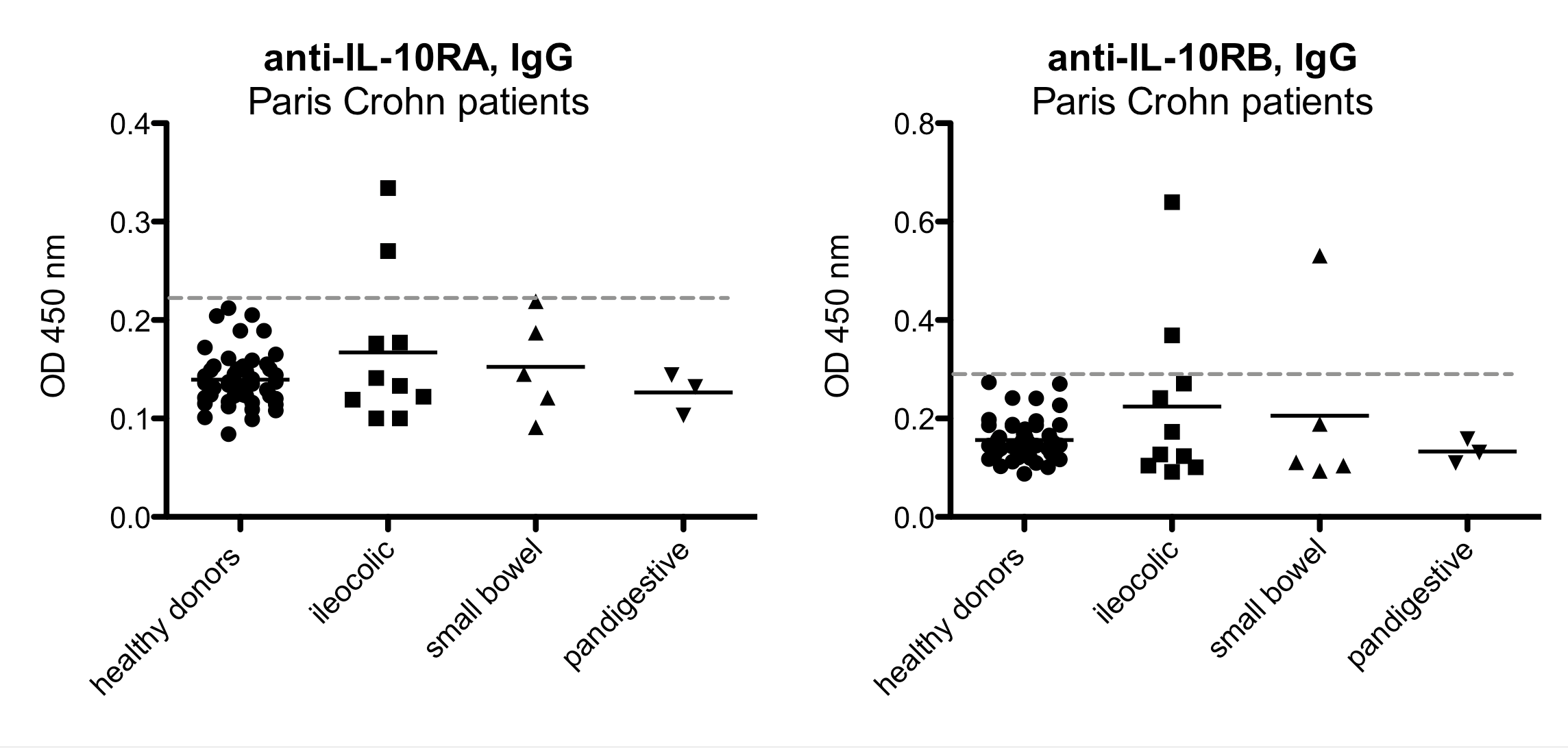


C

Supplement: Additional file 1: Figure S1 — Subgroup analysis of Crohn’s disease patients from Paris. Crohn’s disease patients were subgrouped for disease localisation after their sera were tested for IgG (A) and IgA (B) autoantibodies against IL-10 and IgG autoantibodies against the IL-10 receptor (C) by ELISA. The dotted line represents the cut-off, whereas means are depicted as solid lines. [file 1471-2172-15-10-S1.doc]
